# Polar front associated variation in prokaryotic community structure in Arctic shelf seafloor

**DOI:** 10.3389/fmicb.2015.00017

**Published:** 2015-01-23

**Authors:** Tan T. Nguyen, Bjarne Landfald

**Affiliations:** ^1^Centre for Research-based Innovation on Marine Bioactives and Drug Discovery (MabCent-SFI), UiT The Arctic University of NorwayTromsø, Norway; ^2^Faculty of Biosciences, Fisheries and Economics, Norwegian College of Fishery Science, UiT The Arctic University of NorwayTromsø, Norway

**Keywords:** archaea, bacteria, Barents Sea, beta-diversity, sediment, 16S rRNA gene

## Abstract

Spatial variations in composition of marine microbial communities and its causes have largely been disclosed in studies comprising rather large environmental and spatial differences. In the present study, we explored if a moderate but temporally permanent climatic division within a contiguous arctic shelf seafloor was traceable in the diversity patterns of its bacterial and archaeal communities. Soft bottom sediment samples were collected at 10 geographical locations, spanning spatial distances of up to 640 km, transecting the oceanic polar front in the Barents Sea. The northern sampling sites were generally colder, less saline, shallower, and showed higher concentrations of freshly sedimented phytopigments compared to the southern study locations. Sampling sites depicted low variation in relative abundances of taxa at class level, with persistent numerical dominance by lineages of Gamma- and Deltaproteobacteria (57–66% of bacterial sequence reads). The Archaea, which constituted 0.7–1.8% of 16S rRNA gene copy numbers in the sediment, were overwhelmingly (85.8%) affiliated with the Thaumarchaeota. Beta-diversity analyses showed the environmental variations throughout the sampling range to have a stronger impact on the structuring of both the bacterial and archaeal communities than spatial effects. While bacterial communities were significantly influenced by the combined effect of several weakly selective environmental differences, including temperature, archaeal communities appeared to be more uniquely structured by the level of freshly sedimented phytopigments.

## Introduction

Microbial community similarities tend to show a distance decay relationship, implying that the phylogenetic composition of communities becomes increasingly dissimilar with increasing geographical distance. It is now generally accepted that both contemporary environmental parameters and historical contingencies, maintained by dispersal limitation, may contribute to this beta-diversity. Hence, the classical Baas Becking statement “Everything is everywhere, but the environment selects” is questioned as a universal model for explaining the observed variation in microbial community composition (Hedlund and Staley, 2003; Martiny et al., 2006; Hanson et al., 2012). In the conceptual framework of metacommunity ecology (Leibold et al., 2004; Logue and Lindström, 2008) this emphasis on local environmental factors vs. spatial (regional) effects largely coincides with the distinction between species sorting and mass effects as the two models best explaining microbial community assembly dynamics (Lindström and Langenheder, 2012). The disentanglement of these different effects is, however, not trivial in many systems due to spatial autocorrelation or co-variations among environmental variables (Horner-Devine et al., 2004; Böer et al., 2009; Zinger et al., 2011; Bienhold et al., 2012; Jacob et al., 2013; Wang et al., 2013).

The microbial communities in the upper sediment layers in marine environments show a steeper decay in similarity with distance than assemblies of the pelagic water masses, which may be attributed to more pronounced environmental gradients within the sediments and more restricted dispersal of sediment microorganisms. Additionally, the more heterogeneous environments in coastal areas have been found to generate steeper gradients than such found in the open ocean both in the seawater and sediments (Zinger et al., 2014). The environmental conditions on the continental shelf seafloors may in several respects be characterized as intermediate between those of the deep ocean and the shallow coastal areas. Due to the combination of less water depth and frequently much higher primary production than in the open oceans, the shelf sediments will receive higher influxes of sedimentary material (Suess, 1980) that sustain stronger heterotrophic activity. The bottom-dwelling fauna, including bioturbating animals (Bertics and Ziebis, 2009) and demersal fishes, contributes to resuspension of sediment particles into the water column, as anthropogenic influences (e.g., from bottom trawling) may do as well. Moreover, because ocean currents, including tidal currents, have often been found to be of great importance at the shelf seafloors, microorganisms are likely dispersed quite efficiently, thereby making mass effects a potentially import factor in the establishment of microbial community assemblies in this habitat type.

The Barents Sea (1.4 mill km^2^) is part of the circumpolar Arctic Continental Shelf. It extends northwards from the northern coasts of Norway and Russia to the Arctic Ocean, and is delimited by the Novaya Zemlya and the Norwegian Sea along the east-west axis. With an average depth of 230 m, it is the deepest of the Arctic shelf seas. It is also characterized by less coastal erosion and river water inflow than other Arctic shelf seas (Vetrov and Romankevich, 2004). The most distinctive oceanographic feature of the Barents Sea is, however, the influx of temperate and salty Atlantic water from the southwest. These water masses meet and mix with sub-zero, less saline Arctic Ocean water from the north, resulting in a coarse division of the Barents Sea into a northern and a southern region separated by a transition zone named the polar front (Ingvaldsen and Loeng, 2009). The temperature differences are most pronounced in the surface waters, resulting in winter sea ice covering the northern regions, while the southern parts of the Barents Sea are ice-free throughout the entire year. Near the seafloor, the temperature difference is modest, i.e., about 2°C, and it has even shown a diminishing trend in recent years (Lind and Ingvaldsen, 2012). The overall primary production is highest in the southern parts (Sakshaug et al., 2009) but the deposition of organic material shows a more patchy pattern, caused by additional factors like water depth, bottom topography and local currents (Vetrov and Romankevich, 2004).

On this background, the primary aim of present study was to explore if significant community variations could be detected in a sampling area, which encompassed the moderate environmental variations of the Barents Sea polar front. And if such variations were detectable, should they be attributed to environmental or spatial effects, or both. Beta-diversity analyses were based on 16S rRNA gene sequence data obtained by 454 pyrosequencing. Additionally, the study provided a comprehensive picture of the prokaryotic alpha-diversities in the upper centimeters of this kind of arctic shelf seafloor.

## Materials and methods

### Sampling

**Figure 1:**
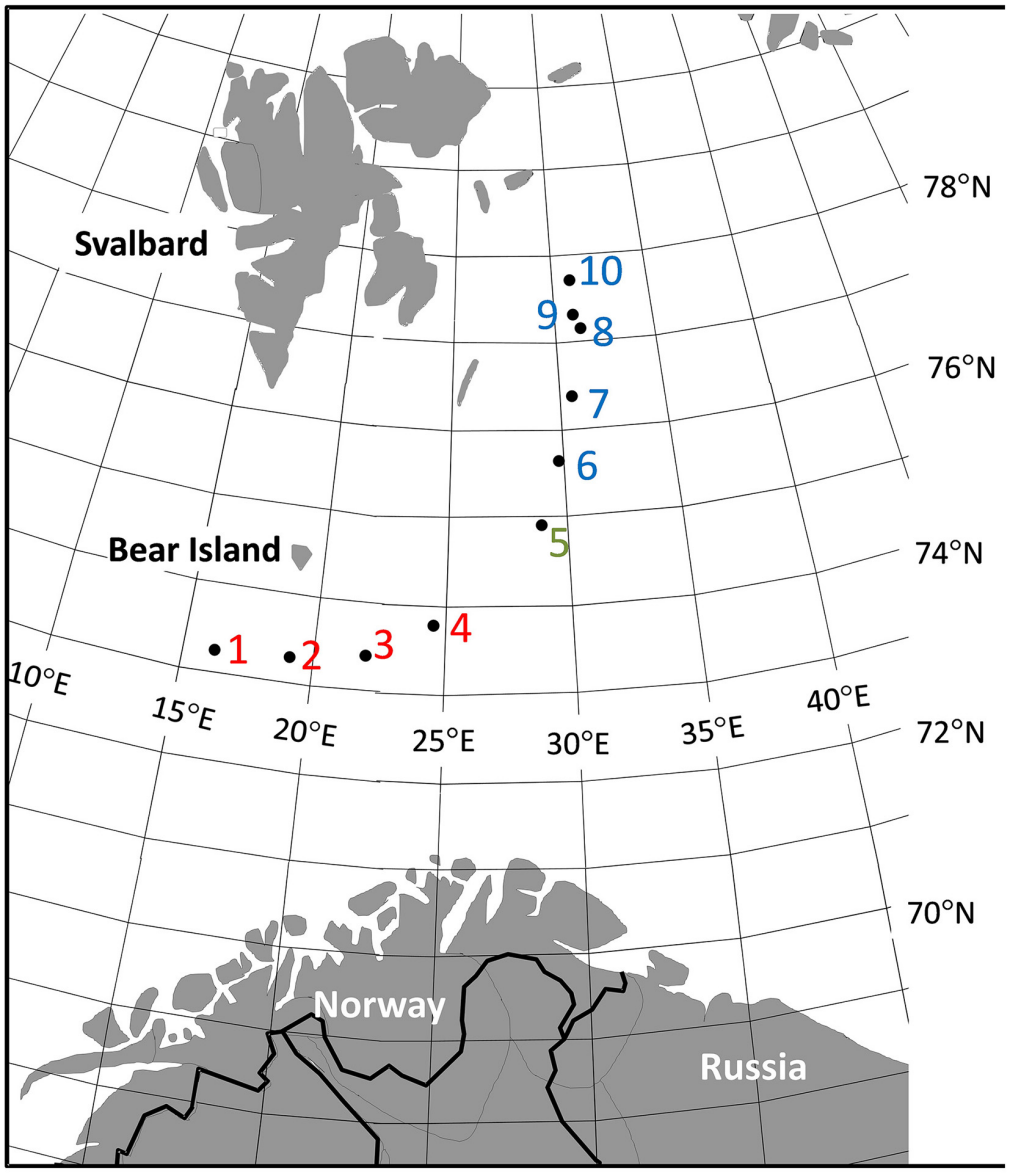
**The western Barents Sea with geographical positions of sampling stations**. Red color (southern part); blue color (northern part), green color (transition temperature zone).

Sediment samples were taken from 10 locations in the western Barents Sea separated by up to 640 km. Sampling was carried out over the course of 3 days from 20th to 23rd May 2009. The sampling was done along a curved transect that followed the gradually more shallow Bear Island – Hopen channel from close to the continental slope to east of the Svalbard archipelago (Figure 1). Seawater temperature and salinity, as measured within 10 m of the seafloor by a CTD instrument, were used as proxies for seafloor values. The upper 4 cm sediment cores of van Veen grab samples were pressed into sterile plastic tubes. The content of each core was homogenized by mixing and stored frozen at -80°C until processing in the laboratory.

### Sediment characteristics

Grain size distribution was determined by dry sieving. The sediment samples were separated into two grain size classes, i.e., clay/silt (<63 μm) and sand/gravel (>63 μm). Total organic carbon (TOC) content was analyzed by a LECO CS-200 Analyzer (LECO Corporation, St. Joseph, MI, USA). Sediment chlorophyll *a* (Chl *a*) and phaeophytin were determined by a Turner 7000 fluorometer (Turner Designs Inc., Synnyvale, CA, USA) from readings at 665 nm in ethanol extracts before and after treatment with 1 M acetic acid (Pápista et al., 2002).

### DNA extraction

Total DNA was extracted from duplicate 0.5 g samples of each site using the PowerSoil™ DNA Isolation kit (Mo Bio Labs, Inc., Carlsbad, CA, USA) according to the manufacturer's instructions. The concentration and quality of extracted DNA were determined by a NanoDrop ND-1000 spectrophotometer (Thermo Scientific, Wilmington, DE, USA).

### Bacterial and archaeal abundances

Quantification of 16S ribosomal RNA genes was used for the estimation of prokaryotic cell densities. Quantitative real-time PCR (qPCR) was performed on an ABI 7500Fast real-time PCR system (Applied Biosystems, NYSE, Waltham, MA, USA) using primers 27F/338R for Bacteria and A571F/915R for Archaea (see Supplementary Table S1). The environmental DNA samples were run in duplicate with three dilutions of the primary extract (10^−1^ to 10^−3^). Standard curves for threshold cycle (Ct) vs. logarithm of the start concentration of 16S rRNA gene copies, from 10^6^ to 10^1^, were established with *Escherichia coli* K10 for Bacteria and *Methanoplanus petrolearius* DSM11571 for Archaea. This corresponded to *E. coli* genomic DNA being serially diluted from 0.76 to 0.76 × 10^−5^ ng and *M. petrolearius* diluted from 1.56 to 1.56 × 10^−5^ ng. Genomic standards were included in each qPCR run to ensure linearity and expected slope values of the Ct/log[gDNA] curves.

### Amplification and multiplex pyrosequencing of 16S rRNA genes

Tagged PCR primers for each sampling station were constructed by adding unique oligonucleotides to the universal forward primers 27F for Bacteria and 571F for Archaea (Supplementary Table S1). The 25 μL PCR reaction mixtures contained 1X PCR buffer (Invitrogen, Waltham, MA, USA), 0.2 mM dNTPs (Invitrogen), 0.5 μM of each primer (Eurofins MWG, Ebersberg, Germany), 1.25 U of *Taq* polymerase (Invitrogen), and 10 ng of genomic DNA template. The thermocycler (Applied Biosystems) conditions were: initial denaturation step at 94°C for 5 min; 30 cycles at 94°C for 30 s, 55°C for 30 s, and 72°C for 1 min; a final extension at 72°C for 5 min. To minimize potential random PCR biases, each sample was amplified in sextuplicate (triplicates of each DNA isolation). Correctly sized amplification products were extracted from the gel by use of the QIAquick Gel Extraction kit (Qiagen, Hilden, Germany), and replicate samples were pooled and purified one more time with Agencourt AMPure XP beads (Beckman Coulter, Brea, CA, USA). Equal amounts of amplicons from each PCR run were pooled and subjected to multiplex pyrosequencing using a 454/Roche GS-FLX Titanium instrument (454 Life Sciences, Branford, CT, UAS) installed at the Norwegian High Throughput Sequencing Centre (NSC, Oslo, Norway; http://www.sequencing.uio.no). The bacterial (BM) and archaeal (AM) amplicons were sequenced separately, as was a second bacterial preparation from sampling station 6 (D6). The latter was subjected to a deeper sequencing effort than used in the multiplex analysis. The raw sequence data have been submitted to the EMBL database under the accession numbers ERP003605 (BM dataset), ERP003606 (AM dataset), and ERP003607 (D6 dataset).

### Sequence analyses

Quality checks, OTU clusterings and phylogenetic annotations of the sequences were all done by the Quantitative Insights Into Microbial Ecology (QIIME v.1.8.0) pipeline (Caporaso et al., 2010b). In brief, low quality sequences were removed, including sequences shorter than 150 bp or with a quality score below 25. Furthermore, sequences containing ambiguous nucleotides or homopolymers longer than six nucleotides were removed (Huse et al., 2007) using Denoiser software (v.0.91) (Reeder and Knight, 2010). Putative chimeras were identified by ChimeraSlayer and discarded (Haas et al., 2011). The overall numbers of pyrotags were reduced by 26.0% for Bacteria and 12.9% for Archaea by removing low-quality, chimeric and chloroplast-affiliated reads. The qualified sequences were clustered into Operational Taxonomic Units (OTUs) based on 97% sequence similarity by the UCLUST algorithm (Edgar, 2010), and representative sequences from each OTU were aligned to the GreenGenes (version May 2013) public database (http://greengenes.lbl.gov) using the PyNAST tool, as integrated in the QIIME package (DeSantis et al., 2006; Caporaso et al., 2010a). Taxon assignments were obtained with 80% bootstrap cutoffs for both Bacteria and Archaea.

Singletons, i.e., OTUs with only one sequence, were removed as putative sequencing errors or PCR amplification artifacts to prevent artificial diversity inflation (Huse et al., 2010; Kunin et al., 2010). The singletons constituted 62.8 and 38.2% of the primary bacterial and archaeal datasets, respectively. OTU richness was calculated by the non-parametric Chao1 estimator (Chao, 1984) after normalization of the sequence numbers in each sample to 4000 for the Bacteria and 9000 for the Archaea.

### Statistical analyses

A geographical distance matrix was calculated from the latitude and longitude coordinates obtained by the Global Positioning System by use of the package *fossil* (Vavrek, 2011) in the R statistical software (R Development Core Team, 2008). The community beta-diversities were determined by the Bray-Curtis, Sørensen and phylogenetic distance based unweighted UniFrac indices, as implemented in the QIIME and R software packages (Lozupone et al., 2006; R Development Core Team, 2008). The community distance matrices were based on jackknifing (100 permutations) with 75% of the sequence number in the sample with the lowest number of sequences.

To visualize the grouping patterns of the samples based on community distances, non-metric multidimensional scaling (NMDS) based on the Hellinger transformed Bray-Curtis distance metric was used (Legendre and Gallagher, 2001). Vector fitting was employed to identify directions and strengths of the effects of environmental factors and geographical distance in relation to the community-based ordination of samples, in accordance with the procedure of Monier et al. (2014). This included the use of the *envfit* function of the *vegan* package in R (Oksanen et al., 2012).

The combinations of environmental variables that best explained community variation among the sampling stations were obtained as the ones generating maximum rank correlations between the environmental and community distance matrices (Clarke and Ainsworth, 1993) by employing the *bioenv* procedure in the *vegan* R package. Generalized linear models (GLM) were subsequently constructed in R from the standardized environmental variables to quantify their relative importance and test the significance of the individual environmental factors by using the *glm* function. To partition the possible community structuring effects of geography and environmental factors, partial Mantel tests were used (Legendre and Legendre, 1998; Martiny et al., 2011). To test if southern and northern communities were significantly different, a multivariate generalized linear models approach (Warton et al., 2012) was employed as implemented in the R package *mvabund* (Wang et al., 2012). The model that was fitted is log-linear and assumes a negative binomial distribution of data. To determine which taxa contributed the most to the differences between the two regions, the univariate ANOVA function with adjusted *p*-values for multiple testing in *mvabund* was used. Community distance decays were calculated by regressing the community distance matrices on the geographical distance matrices. The significance of these decays was determined by simple Mantel tests based on Spearman rank correlation coefficients (ρ) with 10^4^ Monte Carlo permutations. The same procedure was used for testing the relationships between geographical and environmental distances. Tests for correlations between bacterial and archaeal abundances and environmental variables were also based on Spearman rank correlation coefficients.

## Results

### Environmental variation and prokaryotic abundances

Sediment samples from 10 stations separated by up to 640 km were collected during a time period of 3 days, implying that the impact of temporal changes due to the length of the sampling period was minimized. The temperature recordings through the sampling area confirmed a consistent drop of roughly 1.6°C at the seafloor, when moving from the southern stations (1–4) to the northern ones (6–10), while station 5 was in a transitional temperature zone (Table 1). The temperature variation showed significant spatial autocorrelation (Spearman ρ = 0.87; *p* = 0.001), as did the additional environmental factors water depth (Spearman ρ = 0.87; *p* = 0.001), salinity (Spearman ρ = 0.39; *p* = 0.03), and Chl *a*/phaeophytin ratio (Spearman ρ = 0.41; *p* = 0.02), the latter being used as indicator of freshly sedimented phytopigment material. On the other hand, the grain size distribution and organic content of the sediment showed a more random variation between the sampling stations. Principal component ordination, based on the environmental data, separated the sampling stations in accordance with the south-north dichotomy along PC1 (Figure S1 in the Supplementary Information). Noticeably, the peak phytopigment concentration at station 6 was reflected in the fraction of the putative chloroplast 16S rRNA gene sequence reads to the total sequence reads, which also showed a distinct maximum at station 6 (Table 1).

**Table 1:** **Geographical locations and environmental characteristics of samples**.

**St**	**Latitude (N)**	**Longitude (E)**	**Depth (m)**	**Temp (°C)**	**TOC (%)**	**Clay/silt (%)**	**Salinity (μg/gdw^−1^)**	**Chl*a***	**Chl*a*:Phae ratio**	**Chl-16S (%)**	**16S gene copies/g**	
											**Bacteria (×10^9^)**	**Archaea (×10^7^)**
1	73°13′52″	16°20′55″	474	2.7	0.73	67.2	35.01	0.96	0.33	0.1	5.4 ± 0.3	9.7 ± 3.4
2	73°17′74″	19°15′59″	460	2.8	2.24	38.7	35.05	0.55	0.42	0.1	3.1 ± 0.5	6.9 ± 2.4
3	73°23′99″	22°03′13″	450	2.6	2.44	77.0	35.06	1.04	0.32	0.0	4.5 ± 0.2	5.6 ± 0.9
4	73°47′55″	24°35′38″	442	2.5	1.61	86.0	35.05	1.13	0.32	0.2	10.7 ± 3.2	19.0 ± 6.6
5	74°55′01″	28°54′52″	364	1.7	1.96	86.9	35.05	3.24	0.54	1.0	10.8 ± 1.0	7.9 ± 0.7
6	75°38′81″	29°44′48″	330	1.1	2.21	83.5	35.04	8.10	0.96	8.3	13.7 ± 0.8	12.8 ± 5.3
7	76°24′12″	30°37′13″	290	1.2	1.83	86.8	34.98	3.91	0.77	2.5	17.0 ± 0.5	26.7 ± 2.5
8	77°08′92″	31°16′67″	189	1.1	1.21	63.5	34.99	2.00	0.53	2.0	10.2 ± 0.9	8.2 ± 3.5
9	77°20′48″	30°58′81″	194	1.2	1.21	63.5	34.97	1.92	0.64	0.3	11.5 ± 1.6	18.5 ± 1.4
10	77°43′10″	30°56′30″	230	0.9	1.26	66.1	34.97	3.67	0.97	1.8	8.8 ± 0.4	9.4 ± 0.0

*Abbreviations: St, station; Temp, temperature; TOC, total organic carbon in % of dry weight; Chla, chlorophyll a; Phae, phaeophytin; Chl-16S, chloroplast 16S rRNA genes as % of total sequence reads in each sample*.

Bacterial 16S rRNA gene copy numbers varied in the range of 3.1 × 10^9^ to 1.7 × 10^10^ per g dry sediment, and the Archaea constituted 0.7 to 1.8% of total 16S rRNA copy numbers in the corresponding samples (Table 1). If employing the empirical average rRNA operon numbers of 3.9 for Bacteria and 1.8 for Archaea (Lee et al., 2009), the quantitative PCR figures corresponded to 7.9 × 10^8^ to 4.4 × 10^9^ bacterial cells per g and 3.2 × 10^7^ to 1.5 × 10^8^ archaeal cells per g, respectively. A positive correlation was observed between the bacterial and archaeal copy number log abundance values (Spearman ρ = 0.75; *p* = 0.01). Furthermore, the bacterial gene abundance showed significant relationships with temperature (Spearman ρ = 0.60; *p* = 0.04), and phytopigment ratio (Spearman ρ = 0.66; *p* = 0.04), while no correlations were found between the abundance and environmental data for the Archaea.

### Prokaryotic diversity

**Figure 2:**
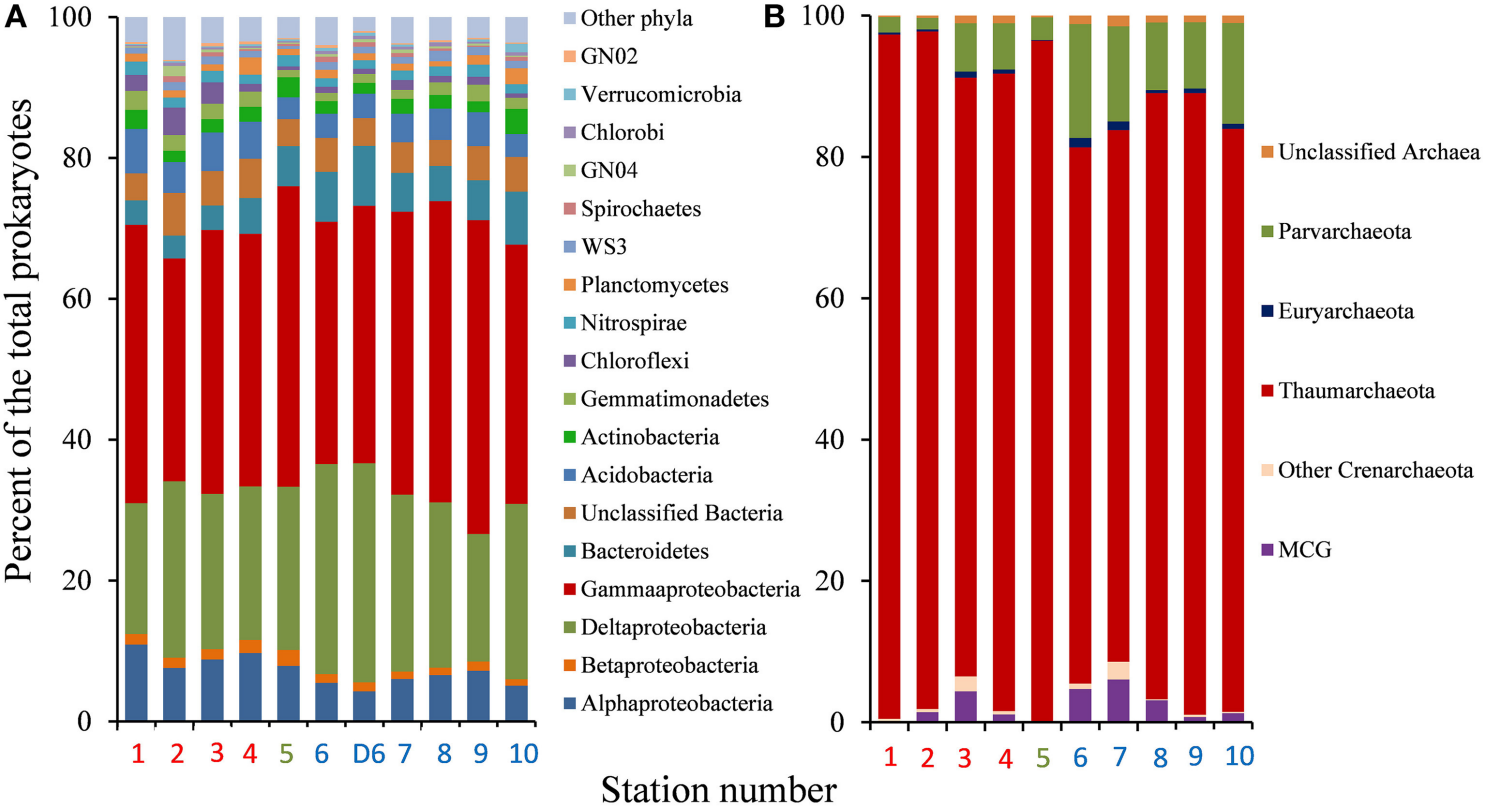
**Distribution of major phylogenetic groups of Bacteria (A) and Archaea (B) at each sampling station**. The analyses of both the multiplex (BM) and the deeper D6 pyrotag datasets are presented for Bacteria at station 6. Abbreviations: MCG, Miscellaneous Crenarchaeotal Group. “Other Crenarchaeota” include Marine benthic group A, Marine benthic group B and Marine Hydrothermal Vent group. Red color (southern part), blue color (northern part), green color (transition temperature zone).

The sequence datasets comprised 65 904, 139 590, and 164 880 qualified reads (excluding reads representing singletons) from the sequencing of the bacterial (BM) and archaeal (AM) multiplex amplicons of the transect and a deeper bacterial sequencing of station 6 (D6). A high bacterial diversity was confirmed in this Barents Sea sediment sample as the numbers of unique OTUs obtained both from the multiplex BM and the single station D6 material exceeded 5500 at ≥97% sequence identity (Table 2). The 21-fold deeper D6 sequencing of station 6, as compared with the BM data, led to a more than four-fold increase in the OTU richness estimate for this station by the Chao1 estimator. This suggested that deeper sequencing of all stations would result in corresponding increases in richness estimates as observed for station 6. Proteobacteria were shown to be dominant in the Barents Sea seafloor by comprising an average of 73.8% of bacterial sequence tags (Figure 2A). Gammaproteobacteria and Deltaproteobacteria accounted for 41.1 and 23.2% of the reads, respectively. The taxonomic assignment pointed to the Piscirickettsiaceae as the most prominent sub-group of the Gammaproteobacteria (43.6% of sequence reads), while a substantial fraction of the deltaproteobacterial reads (31.9%) were affiliated with the orders Desulfobacterales and Desulfuromonadales.

**Table 2:** **Pyrosequencing statistics, number of operational taxonomic units at 97% similarity level and richness estimates**.

**St**	**Bacteria**			**Archaea**		
	**Qualified reads**	**OTUs**	Chao1^*^	**Qualified reads**	**OTUs**	Chao1^*^
1	6148	1799	2842	12,023	253	369
2	6148	1856	2728	13,613	266	343
3	4033	1583	3593	18,502	695	883
4	11,417	2872	3542	18,510	585	594
5	5387	1604	2963	9006	209	353
6	7761	2116	3339	11,821	805	1044
7	7049	2096	3392	18,271	972	1184
8	5157	1578	2918	10,704	492	659
9	8238	2263	3064	14,410	578	696
10	4566	1578	3369	12,730	650	876
D6	164,880	9072	14,016			

*Abbreviations: St, Station; OTUs, operational taxonomic units. D6; Deeper sequence at station 6*.

* *Computed on quality read subsampled at an even depth of 4000 sequences for bacteria and 9000 sequences for archaea*.

Despite the more than two-fold deeper sequencing of the AM than the BM dataset, archaeal OTU numbers were, on average, 29% of the bacterial figures for the same stations. The archaeal communities were highly dominated by a few phylotypes, as the three most prevalent OTUs constituted 60 to 89% of total sequence reads in the different samples. The other striking feature of the archaeal communities was the overwhelming quantitative dominance by the class Thaumarchaeota, which averaged 85.8% of archaeal sequence reads in the samples (Figure 2B). A substantial fraction (33.9%) of the thaumarchaeotal reads was affiliated with the marine, ammonia-oxidizing genus *Nitrosopumilus*. Besides the Thaumarchaeota, phylotypes representing the Miscellaneous Crenarchaeotal Group (Inagaki et al., 2003), the Marine benthic group B (Knittel et al., 2005) and the candidate phylum Parvarchaeota (Rinke et al., 2013; Hedlund et al., 2014) constituted significant groups, while less than 1% of the archaeal sequence reads showed euryarchaeotal affiliation.

### Community structure variation

**Figure 3:**
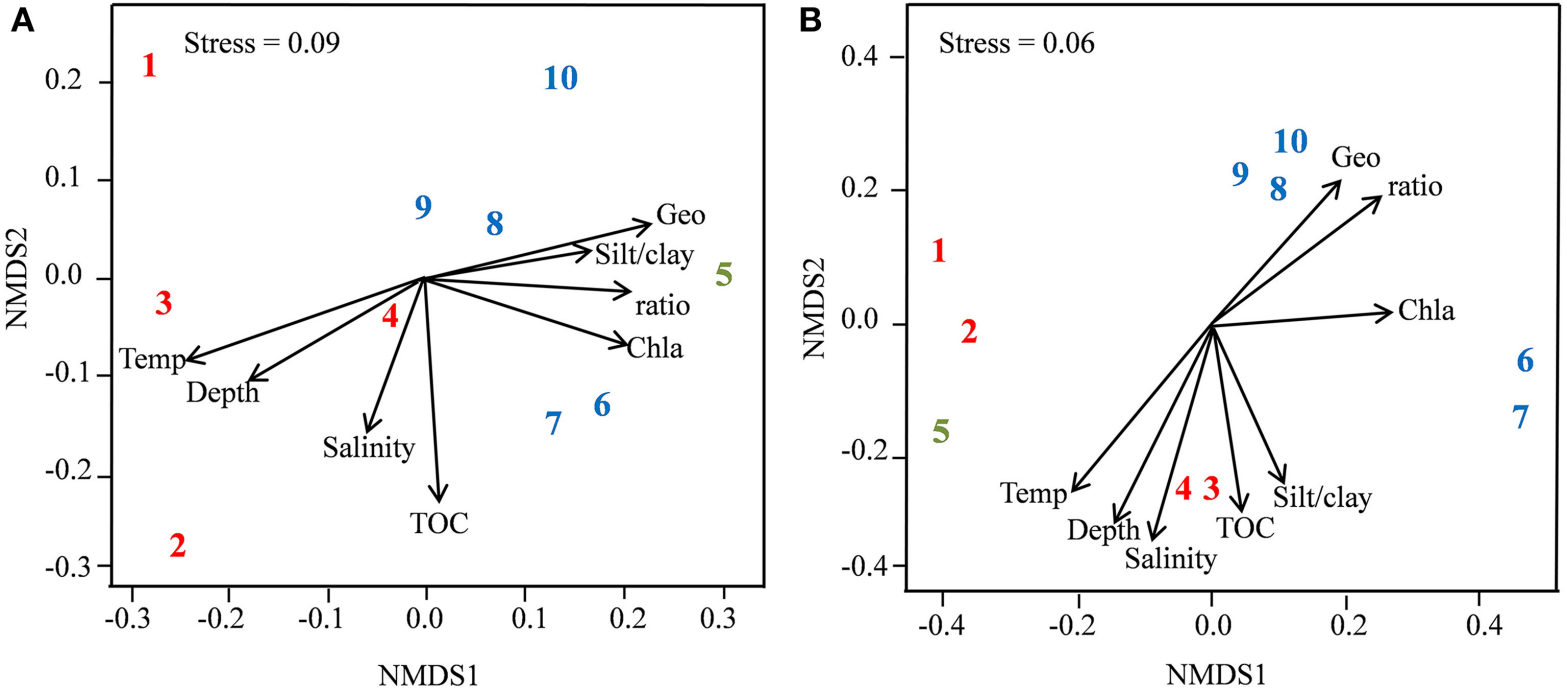
**Non-metric multidimensional scaling based on Bray Curtis community distances for Bacteria (A) and Archaea (B)**. Numbers represent sampling stations and arrows show vector fitting of the environmental variables. Abbreviations: Temp, Temperature; Depth, water depth; TOC, total organic carbon; Chl *a*, chlorophyll *a*; ratio, chlorophyll a/phaeophytin ratio; Geo, spatial distance between sampling stations. Red color (southern part), blue color (northern part), green color (transition temperature zone).

**Figure 4:**
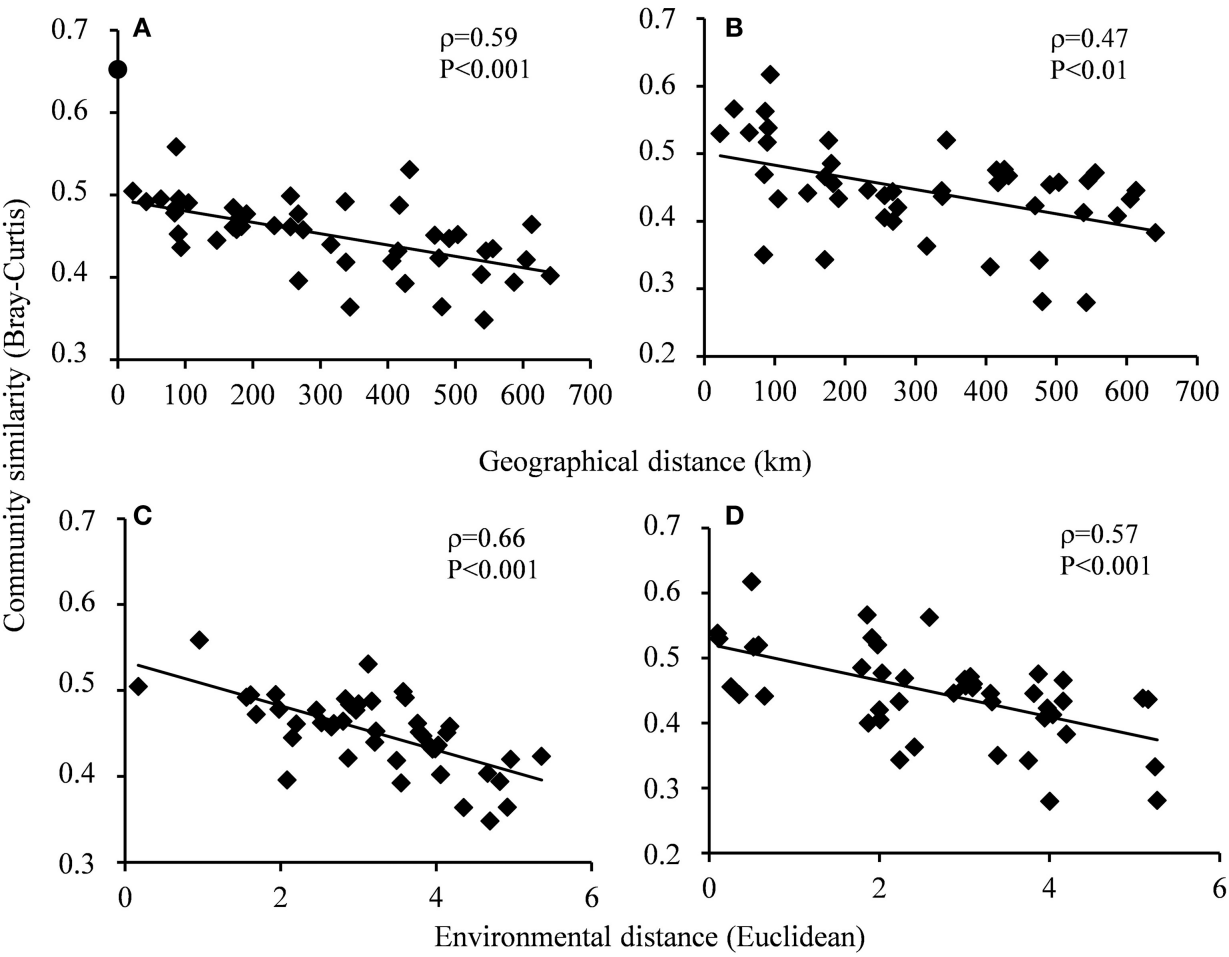
**Relationships between community similarity (1-Bray Curtis index) and spatial distance (A,B) and between community similarity and environmental distance (C,D) for Bacteria (A,C) and Archaea (B,D)**. The beta-diversity of the two bacterial station 6 datasets (filled circle in **A**) is not included in the regression line or correlation analyses. The significance of the correlations were assessed by Mantel tests based on Spearman's rank correlation with 10^4^ Monte Carlo permutations.

The overall stable distribution of phylotypes (Figure 2) and congruent ranking of abundant OTUs (data not shown) at the different sampling stations for both the Bacteria and Archaea weighed against strong community structuring forces within this range of arctic seafloor. However, NMDS ordination based on the complete sequence information (singletons not included) indicated some level of clustering of the prokaryotic communities in accordance with the separation by environmental factors and spatial distance. For both Bacteria and Archaea, the communities of stations 1–4 tended to be associated with the slightly warmer, deeper and more saline conditions in the southern part of the sampling range, while the communities from stations 6–10 were associated with the observed higher levels of the phytopigment indicators in that region (Figure 3). Statistical comparisons between the southern (1–4) and northern (6–10) communities showed significant differences by the multivariate generalized linear models approach for Bacteria and Archaea (ANOVA, *p* = 0.009 for both groups). The five bacterial taxa that generated most explained difference between the two regions were the proteobacterial orders Nitrosomonadales (*p* = 0.006), Rhodospirillales (*p* = 0.009), Marinicellales (*p* = 0.009), Desulfuromonadales (*p* = 0.010) and the uncultured proteobacterial group Sva0853 (*p* = 0.010). For the Archaea, just the variation in the Parvarchaeota (*p* = 0.001) and Thaumarchaeota (*p* = 0.021) tag abundances gave significant contributions to explained regional difference (Supplementary Figures S2, S3). Distance decays of community similarity were confirmed both by the Bray-Curtis index (Figures 4A,B) and the phylogeny-based unweighted UniFrac dissimilarity index (ρ = 0.34; *p* = 0.02 for Bacteria; ρ = 0.54; *p* = 0.01 for Archaea). The zero-distance bacterial Bray-Curtis index value of 0.65 (Figure 4A) represents the similarity between the BM and the D6 sequence pools of station 6. They constituted separate, independently analyzed DNA extracts from the same, well-mixed sediment sample material. Hence, the deviation of this value from unity reflects the stochastic beta-diversity associated with non-exhaustive sequencing within a single community. The |β| coefficients, i.e., the absolute values of the linear regression coefficients based on the Sørensen similarity index in a double logarithmic plot (Zinger et al., 2014) were 0.056 ± 0.013 for the Bacteria and 0.153 ± 0.041 for the Archaea.

Mantel tests showed that the independence of possible influential factors on the community structuring was obscured both by significant collinearities between several of the individual environmental factors, i.e., temperature, depth, salinity and phytopigment ratio (*p* = 0.001 for all combinations) and by spatial autocorrelations of the same environmental factors (*p* = 0.001). Hence, relationships were optimized between combinations of environmental parameters and community variation by the *bioenv* procedure, and significant relationships were found between these combinations of environmental factors and community distances (Figures 4C,D). For the Bacteria, the four variables temperature, phytopigment ratio, %silt/clay and TOC were maintained in the model, while depth, phytopigment ratio and Chl *a* gave positive contributions for the Archaea.

Partial Mantel tests were employed to assess the independent effects of space and environment on the community structuring. Significant relationships between community and environmental variation were confirmed when controlling for spatial distance (Table 3). For the reciprocal tests, i.e., spatial effects when controlling for environmental distance, the null hypothesis could not be rejected, but the similar magnitudes of the correlation coefficients for the two bacterial tests indicated comparable contributions to explained variation by environment and space. To quantitate the relative contributions to explained community variation by the different environmental factors, general linear models were established. These models retained the independently varying factors temperature, %silt + clay and TOC as statistically significant contributors for the Bacteria, while the phytopigment ratio alone showed significance for the Archaea. The contribution to overall community variation explained by environmental variables was 42.1% for the bacterial communities and 31.0% for the archaeal communities (Table 4).

**Table 3:** **Partial Mantel tests of Spearman's rank correlations between prokaryotic community distance and either geographical or environmental distance**.

**Correlation between Prokaryotic community and**	**Controlling for**	**Bacteria**		**Archaea**	
		***ρ*-Value**	***p*-Value**	***ρ*-Value**	***p*-Value**
Geographic distance	Environmental distance	0.28	0.06	0.1	0.27
Environmental distance	Geographic distance	0.3	**0.04**	0.43	**0.005**

*Statistically significant relationships are indicated by bold letters*.

**Table 4:** **General linear model analyses of the effect of individual environmental variables on bacterial and archaeal communities**.

	**Coefficient**	***p*-Value**
**BACTERIA**		
Temperature	0.020	**0.009**
TOC	0.016	**0.016**
%(Silt/Clay)	0.014	**0.019**
Phytopigment	0.007	0.432
**ARCHAEA**		
Phytopigment	0.033	**0.024**
Chl-*a*	0.014	0.201
Depth	0.017	0.199

*Statistically significant relationships are indicated by bold letters; TOC, total organic carbon in % of dry weight; Chl a, chlorophyll a; Phytopigment, chlorophyll a/phaeophytin ratio. R^2^ adj = 0.42 for bacteria, R^2^ adj = 0.31 for archaea*.

## Discussion

### Prokaryotic communities in the Barents Sea sediment

Our data confirmed the initial finding of Torsvik et al. (1996) that upper marine sediments harbor one of nature's most diverse microbiotas. High 16S rRNA gene diversity estimates for sediments have previously been obtained from rarefaction analyses of clone libraries (Ravenschlag et al., 2001; Pedrós-Alió, 2006) and, more recently, by massive parallel sequencing efforts (Zinger et al., 2011; Bowen et al., 2012; Hamdan et al., 2013). The four-time increase in the bacterial richness estimate for station 6 when comparing the one based on the roughly 7700 reads of the BM with the 21-fold deeper D6 dataset confirmed the strong dependency on sequencing depth that has previously been documented for the Chao1 estimator (Lemos et al., 2011). As compared with near full-length amplicons of the bacterial 16S rRNA gene, our sequence reads of the V1–V2 region may have produced up to 30% overestimations of OTU richness due to a higher fraction of hypervariable basepairs than in the complete gene (Youssef et al., 2009).

The bacterial taxa composition of the Barents Sea samples was similar to recent reports for marine seafloor upper sediments, i.e., distinctly higher fractions of Deltaproteobacteria, but lower abundances of Alphaproteobacteria than commonly found in the pelagic bacterial communities. This main feature has been observed from deep ocean seabeds with low influx of water-column derived sedimenting material to more shallow coastal areas, where benthic–pelagic coupling likely is strong. It therefore seems to reflect a universal environmental adaptation of the marine sediment bacterial communities (Li et al., 2009; Teske et al., 2011; Zinger et al., 2011; Bienhold et al., 2012). Hence, the uppermost centimeters of the sediment appeared to be dominated by autochtonous bacterial assemblies throughout the sampling area.

The less than 2% of total 16S rRNA genes affiliated with Archaea seems characteristic of the uppermost layer of marine sediments. Comparably low presence of Archaea have been found in other Arctic and Antarctic sediments (Sahm and Berninger, 1998; Ravenschlag et al., 2001; Bowman and McCuaig, 2003). The archaeal communities showed a noticeable skewness in phylotype distribution, as the three top-ranking OTUs constituted more than two-thirds of total archaeal sequence reads and there was an absolute dominance by representatives of the recently established group Thaumarchaeota (Brochier-Armanet et al., 2008). The Thaumarchaeota comprise the phylotypes that were previously classified as Crenarchaeotal Group 1.1a (Schleper and Nicol, 2010), which have been identified as major archaeal constituents in marine pelagic waters and sediments, including polar and other cold regions (Bano et al., 2004; Galand et al., 2009; Dang et al., 2010; Alonso-Sáez et al., 2011; Durbin and Teske, 2011). In contrast, Hamdan et al. (2013) did not identify Thaumarchaeota in the sediment of the Alaska Beaufort Sea shelf. The Thaumarchaeota are associated with an autotrophic ammonia-oxidizing energy metabolism with the capacity to utilize very low substrate concentrations (Könneke et al., 2005; Herfort et al., 2007; Pester et al., 2011). As established ammonia-oxidizing bacterial phylogenetic groups, like Nitrosomonadales, were very poorly represented among the Bacteria, the Thaumarchaeota appeared to be the predominant ammonia oxidizers in this cold shelf sediment. With some reservations regarding seasonal variations or primer bias in the 16S rRNA gene amplification, the virtual absence of relevant groups of Euryarchaeota in our material excluded methanogenesis or anaerobic methane oxidation as significant processes in the top centimeters of this seafloor.

### Sources of community variation

The dissimilarity between spatially separated microbial communities is established in the balance between neutral factors, the rate of dispersal of the organisms and the strength of local selective forces (Sloan et al., 2006; Lindström and Langenheder, 2012; Wang et al., 2013). The Barents Sea comprises a contiguous shelf seafloor, where minor differences in the prokaryotic assemblies were expected due to moderate environmental variations in combination with an anticipated substantial dispersal effected by re-suspension of fine-grained sediment particles. The stability in higher taxa composition throughout the sampling range consolidated this presumption. Additionally, allochtonous influx of microorganisms via particulate pelagic material may have promoted the high community similarity, as the bacterioplankton is more weakly biogeographically structured than the benthic microbiotas across similar distances (Zinger et al., 2014). However, our phylogenetic data gave no basis to conclude that bacterial groups that are associable with sedimenting planktonic material constituted a significant fraction of the seafloor microbiota. The frequently cultivable, copiotrophic lineages of Gammaproteobacteria, principally members of the Alteromonadales, Oceanospirallales, Vibrionales and Pseudomonadales, are pointed out as characteristic of particle-bound planktonic Bacteria (Zhang et al., 2007; Lauro et al., 2011; Teske et al., 2011; Crespo et al., 2013; D'Ambrosio et al., 2014). These groups constituted minor proportions of the Gammaproteobacteria in our sediment material, while representatives of the dominating Piscirickettsiaceae family have not, to our knowledge, been associated with pelagic particulate material.

A main objective of our study was, however, to elucidate if even these small environmental differences across the more than 600 km sampling area transecting the Barents Sea polar front, were reflected in non-random community variations if analyzed by a next-generation sequencing approach. The NMDS ordination patterns of the assemblies of both Bacteria and Archaea suggested some degree of community structuring in accordance with the south-north spatial and environmental separation of the sampling range. However, estimates of the importance of the factors that gave rise to this structuring was complicated both by the extensive collinearity between several environmental factors, i.e., temperature, phytopigment ratio, water depth and salinity, and the just as strong spatial autocorrelation of the same factors. These phenomena weakened the possibility to disentangle the contributions by the various factors to the overall beta-diversity and made general linear models labile, with coefficient estimates that were sensitive to minor changes in the data or the optimization criteria (Legendre, 1993; Dormann et al., 2013).

The spatial separations of sampling sites in the present study varied from 23 to 640 km, thereby falling into the intermediate range (10–1200 km) in which Schauer et al. (2010) have found dispersal limitation and contemporary environmental selective forces to show comparable contributions to biogeographic patterning in deep-sea sediments. We found the impact of spatial effects to be subordinate to the one of environmental factors, although this ranking was less evident for the Bacteria than the Archaea. Hence, the data did not exclude our initial assumption that dispersal is substantial in this kind of shelf sediment, but dispersal was evidently not strong enough to blur the community structuring effects of the moderate environmental differences along the sampling area. The partitioning of the various environmental factors that contributed to explained community variation was based on the criterion of Clarke and Ainsworth (1993) of optimized fit between community and environmentally based distance matrices, in combination with a general linear model. This approach picked two different, covarying environmental variables, i.e., temperature for the Bacteria and phytopigment ratio for the Archaea, as the most influential community structuring factors, with some additional contribution to explained variation by the independently varying factors organic content and grain size distribution for the Bacteria. Published studies in the field or additional data acquired through the present study did not give robust grounds to conclude whether this difference had a true ecological basis or rather was a consequence of model lability caused by collinearity.

The temperature difference between the southern and northern sampling stations constitutes a stable oceanographic feature of the western Barents Sea (Ingvaldsen and Loeng, 2009) but the observed difference appears to be at best marginal with regards to leaving a detectable footprint in the prokaryotic assemblies. Previous documentations of temperature effects have largely been associated with markedly wider ranges (Fuhrman et al., 2008; Gilbert et al., 2009; Wietz et al., 2010; Agogué et al., 2011), while Hamdan et al. (2013) found no contribution to beta-diversity by a ≤2.4°C temperature variation in arctic marine sediment.

At the time of our sampling effort in late May, winter sea ice had retracted from around sampling stations 5–6 to about station 8 and was partly disintegrated even further north. The spring bloom, which is particularly intensive in the 20–50 km marginal zone south of the ice edge (Sakshaug et al., 2009), was well-under way and sedimentation from this bloom may explain the distinctly higher phytopigment and chloroplast-associated 16S rRNA gene levels in the northern part of the sampling range. In addition, the greater water depth in the southern part, with less sedimented material reaching the seafloor, may have contributed in the same direction. Both the Chl *a*/phaeophytin ratio and the concentration of Chl *a* have been used as estimators of freshness of sedimented phytoplanktonic material in e.g., the western Barents Sea. Positive relationships between the content of sediment phytopigments and bacterial growth and production has been demonstrated (Jørgensen and Boetius, 2007; Morata and Renaud, 2008) and pigment content has been used as a proxy for available energy to benthic bacteria in arctic marine sediment (Bienhold et al., 2012). The abundance of Thaumarchaeota in pelagic marine waters has also been shown to correlate positively with Chl *a* (Robidart et al., 2012) but in the present study, the candidate phylum Parvarchaeota rather was the group that showed a marked increase in the northern region. This recently identified group (Rinke et al., 2013) of very small cells with correspondingly small genomes has as yet only been genetically characterized through an acid mine drainage single-cell sequencing project (Hedlund et al., 2014) and these data do not give any hint to its ecological adaptation in marine sediment.

No significant community structuring effects of water depth or salinity were observed. Previous studies documenting effects of water depth are founded on substantially wider depth ranges than the less than 300 m in the present study. In two studies based on pyrosequence data comprising sampling sites from surface level to the deep ocean floor, up to 3.0% of the sediment bacterial community variation was found to be explained by water depth (Zinger et al., 2011; Bienhold et al., 2012). Although consistent, the shift in salinity close to the seafloor between the southern and northern parts of the sampling range was below 0.1%, and we anticipate it generates a negligible structuring effect on the prokaryotic communities.

There is the possibility that unmeasured environmental variables contributed significantly to community variation through the sampling range. Possible unaccounted variables include the levels of inorganic nutrients (Wu et al., 2008; Böer et al., 2009) and the degree of oxygen penetration into the sediment (Durbin and Teske, 2012). However, no variations in abiotic composition have been reported along this well-characterized extent of contiguous soft bottom seafloor, which will likely overshadow the influences by the variables that were included in the study. In addition, the moderate variation in the fraction of taxonomic groups associated with anaerobic sulfur compound metabolisms, e.g., the orders Desulfobacterales and Desulfuromonadales, was not indicative of major changes in oxgen profiles within the upper 4 cm of the seafloor (data not shown).

Several studies have confirmed microbial community distance decay relationships in marine habitat types like pelagic water (Monier et al., 2014; Zinger et al., 2014), salt marshes (Horner-Devine et al., 2004; Martiny et al., 2011), and oceanic sediments (Schauer et al., 2010; Zinger et al., 2014). Sapp et al. (2010) represent an exception as they were unable to detect significant spatially induced variation of bacterial and archaeal communities in North Sea sediment by a denaturing gradient gel electrophoresis approach. The actual magnitudes of the distance decays are, however, difficult to compare due to differences in diversity indices, organismal target groups, genetic entity compared, etc. A recent global sampling study employing an analytical approach highly similar to the one used by us (Zinger et al., 2014) consolidates our estimate of 0.056 for the absolute value of the double-logarithmic distance decay regression coefficient on shelf seafloor. Our figure was in-between the values of for deep-sea and coastal sediments estimated by Zinger et al. (2014). On the other hand, the corresponding coefficient estimated by Schauer et al. (2010) for South Atlantic deep-sea sediments was just 0.003, i.e., at least an order of magnitude smaller than our shelf sea figure, and the authors associate this low distance decay with high dispersal rates and low extinction rates of the vast bacterial populations in this kind of environment. The archaeal coefficient of 0.15 estimated in our study corresponded to the upper extreme bacterial values recorded by Zinger et al. (2014). Together with the above-mentioned assignment of the explained archaeal community variation solely to environmental factors, the archaeal beta-diversity appeared as more sensitive to environmental variation than the one of the Bacteria in this shelf seafloor environment. To our knowledge, this kind of comparative beta-diversity observations between Bacteria and Archaea in the same environment has not previously been reported.

In conclusion, our data consolidated previous findings regarding the bacterial alpha-diversities of marine shelf seafloor sediments but reinforced the significance of the Thaumarchaeota as the principal archaeal group in this type of environment. Furthermore, the study confirmed that biogeographical structures are detectable in marine sediment prokaryotic communities by deep 16S rRNA gene sequencing, even where high dispersal rates combined with weak environmental filtration counteract the build-up of beta-diversity patterns. This may have implications for the practicality of employing such approaches to monitoring microbial effects of e.g., the predicted rise in air and water temperatures in the polar regions, including the Barents Sea, in the years to come. This climate change is expected to be manifested in the microbial communities (Kirchman et al., 2009). On the other hand, the study also emphasized the importance of sufficient prior knowledge of the environmental variations within the sampling area to avoid complications caused by extensive co-variations among the spatial and environmental variables.

### Conflict of interest statement

The authors declare that the research was conducted in the absence of any commercial or financial relationships that could be construed as a potential conflict of interest.
