# Supplementary material for: Polar front associated variation in prokaryotic community structure in Arctic shelf seafloor
Source: Front Microbiol. 2015 Jan 23;6:17. doi: 10.3389/fmicb.2015.00017 (PMC4304239; doi:10.3389/fmicb.2015.00017)
Supplement: Supplementary file 1 [file Table1.PDF]

Supplementary Information

Table S1. Bacteria and Archaea specific 16S rRNA gene sequences used for quantitative PCR and for constructing pyrosequencing primers.

	Primer	Sequences (5'-----3')	References
Bacteria	27F	AGAGTTTGATCMTGGCTCAG	Susuki *et al.*, 1998
	338R	GTGCTGCCTCCCGTAGGAGT	Susuki *et al.*, 1996
Archaea	571F	GCYTAAAGSRICCGTAGC	Baker *et al.*, 2003
	915R	GTGCTCCCCCGCCAATTCCT	Raskin *et al.*,1994

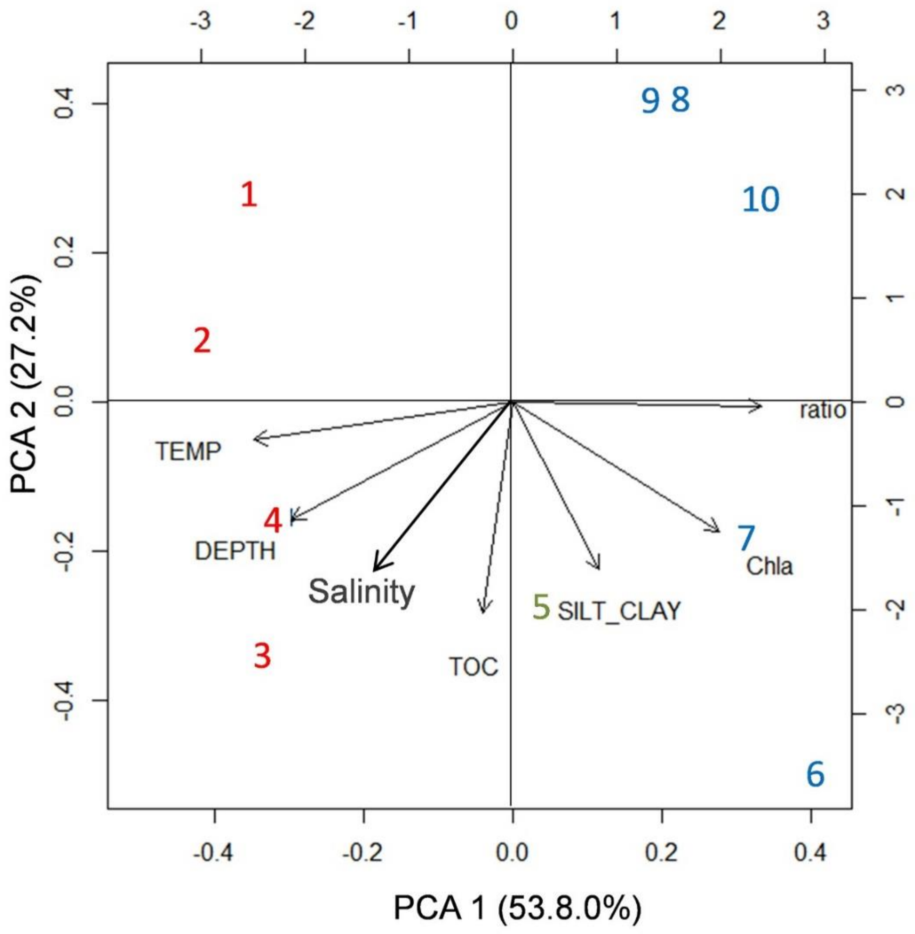

Figure S1. Principal component ordination based on the environmental data. Red colour (southern region); blue colour (northern region), green colour (transition temperature zone).

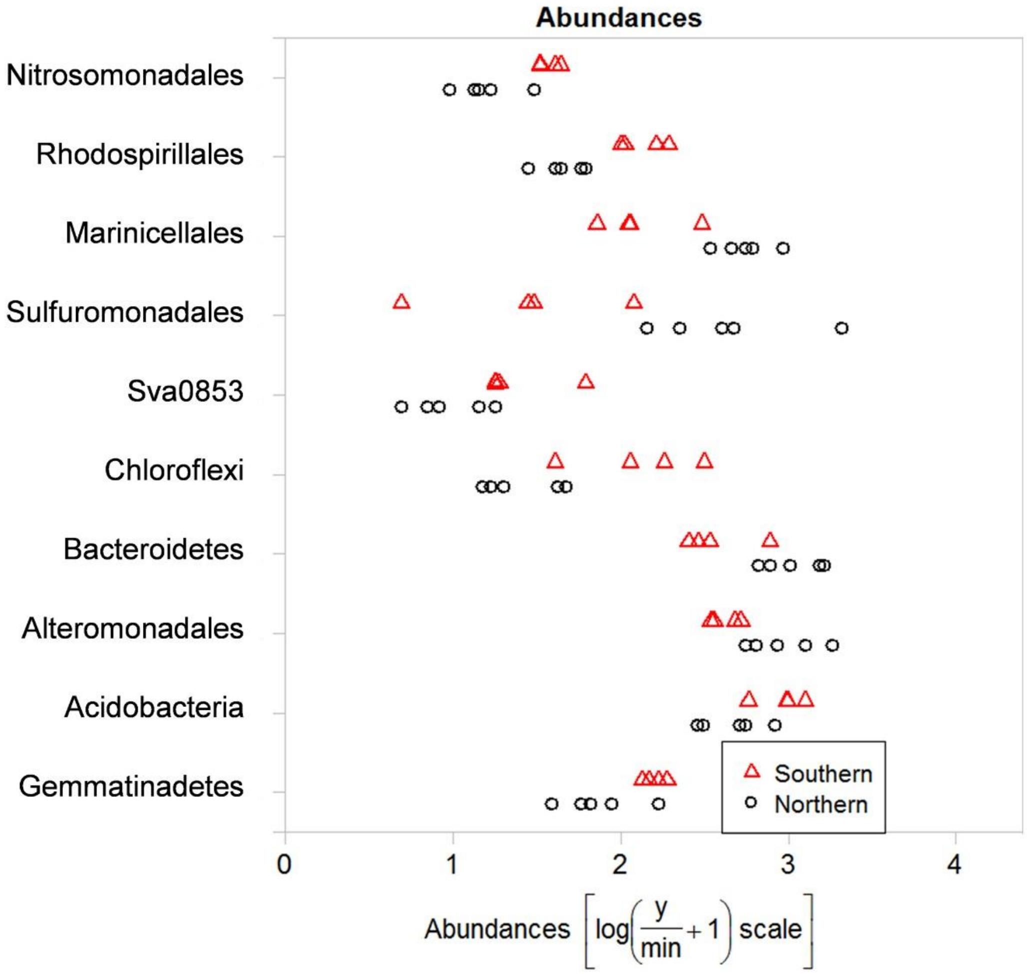

Figure S2. The phyla and orders that contribute the most to the distinction between northern and southern bacterial communities, rank according to their univariate ANOVA p- values.

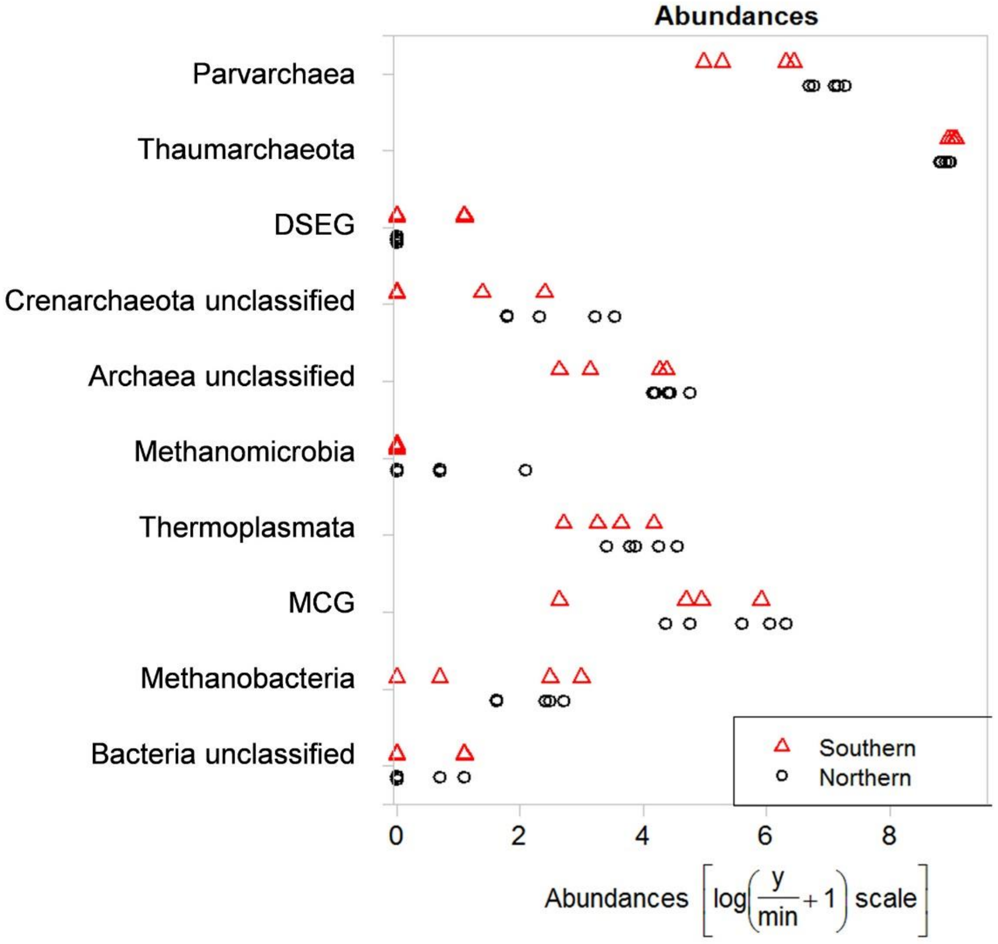

Figure S3. The phyla and classes that contribute the most to the distinction between northern and southern archaeal communities, rank according to their univariate ANOVA p- values.

References

Baker, G. C., Smith, J. J., and Cowan, D. A. (2003). Review and re-analysis of domain-specific 16S primers. *J. Microbiol. Meth.* 55, 541-555. doi: 10.1016/j.mimet.2003.08.009

Raskin, L., Stromley, J.M., Rittmann, B.E., Stahl, D.A. (1994). Group-specific 16S rRNA hybridization probes to describe natural communities of methanogens. *Appl Environ Microbiol* 60:1232-1240.

Suzuki, M.T., Rappe, M. S., and Giovannoni, S. J. (1998). Kinetic bias in estimates of coastal picoplankton community structure obtained by measurements of small-subunit rRNA gene PCR amplicon length heterogeneity. *Appl. Environ. Microbiol.* 64, 4522-4529.

Suzuki, M.T., S.J. Giovanni, S.J. (1996). Bias caused by template annealing in the amplification of mixtures of 16S rRNA genes by PCR. *Appl. Environ. Microbiol.*, 62 pp. 625–630.
